# Retinal Image Simulation of Subjective Refraction Techniques

**DOI:** 10.1371/journal.pone.0150204

**Published:** 2016-03-03

**Authors:** Sara Perches, M. Victoria Collados, Jorge Ares

**Affiliations:** Department of Applied Physics, University of Zaragoza, Zaragoza, Spain; The Ohio State University, Center for Cognitive and Brain Sciences, Center for Cognitive and Behavioral Brain Imaging, UNITED STATES

## Abstract

Refraction techniques make it possible to determine the most appropriate sphero-cylindrical lens prescription to achieve the best possible visual quality. Among these techniques, subjective refraction (i.e., patient’s response-guided refraction) is the most commonly used approach. In this context, this paper’s main goal is to present a simulation software that implements in a virtual manner various subjective-refraction techniques—including Jackson’s Cross-Cylinder test (JCC)—relying all on the observation of computer-generated retinal images. This software has also been used to evaluate visual quality when the JCC test is performed in multifocal-contact-lens wearers. The results reveal this software’s usefulness to simulate the retinal image quality that a particular visual compensation provides. Moreover, it can help to gain a deeper insight and to improve existing refraction techniques and it can be used for simulated training.

## Introduction

Contemporary daily tasks—such as learning, social interaction, or information and communication technology management—require the best visual quality possible. Refraction techniques are devoted to find the most appropriate sphero-cylindrical lens prescription (either spectacles or contact lenses) to achieve the best visual quality possible based on the patient’s visual needs and on specific environmental factors.

In general, objective and subjective refraction are both used in clinical practice. Subjective refraction is a widely-used approach to find the best sphero-cylindrical refractive compensation and is based on the patients’ response to one or several sequential tests [1–3]. In this context, the most commonly applied end-point is that known as “maximum plus to best visual acuity”. Even though subjective refraction is the most popular approach, it is not complication-free. Aside from accommodation interaction, the use of astigmatism tests—such as Jackson’s Cross Cylinder (JCC) test—can be prone to errors [4] and, in the presence of significant high-order aberrations (HOA), the final sphero-cylindrical result can vary depending on the patient’s pupil size [5]. Moreover, young children or patients with mental disability or language difficulties can make the examiner-patient interaction more difficult. As a result, there are people for whom the subjective refraction process does not yield those sphero-cylindrical lenses that lead to the best visual quality possible. Therefore, in order to improve their skills and to make the most of the technique the eye-care professional should train with many patients and over a wide range of situations. Nevertheless, it is difficult for a practitioner to fit regular training into their daily schedule; for this reason, a variety of simulation programs have been developed for subjective-refraction learning [6,7]. Despite this improvement, the truth is that some real-life scenarios, such as the presence high-order aberrations, spectacle magnification or the decision dilemma during Jackson Cross Cylinder test, are not taken into account in these simulation tools.

On the other hand, objective refraction determines the refractive error regardless of the patient’s judgment, relying solely on a set of criteria set in advance by the examiner or by a computer algorithm based on some ocular measurements. Some of these objective-refraction instruments are based on wavefront quality and retinal image quality [8,9]. Among these, one of the most widely-used is the automated refractor [1], which is usually employed in clinical practice as a starting point for subjective-refraction determination. A few simulation software programs have been developed to evaluate these ocular devices, such as the slit retinoscope [10] or the eccentric photorefractor [11].

In this paper, we present a novel simulation software that enables the examiner to calculate and evaluate retinal image quality at each step of the subjective refraction, starting from the patient’s aberrometry assessment [1]. First, a complete description of the software is provided. Then, retinal images corresponding to various subjects undergoing the clock-dial test and Jackson’s Cross Cylinder test are simulated. Finally, this software is also used to predict the potential visual performance of multifocal contact lens designs.

## Material and Methods

**Fig 1:**
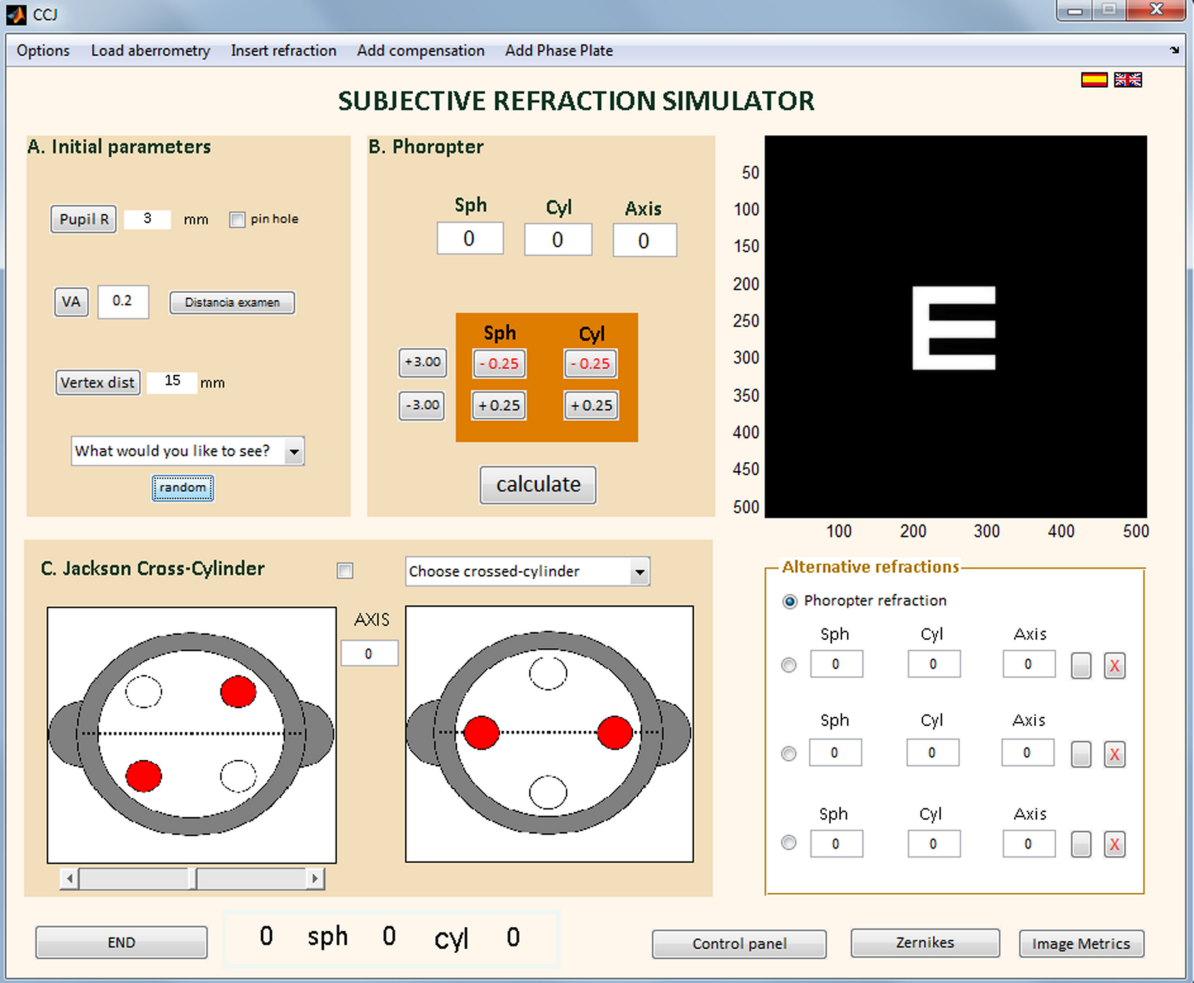
Simulation software. Main screen of the simulation software’s GUI.

This simulation software was written in Matlab v.7 (The Mathworks Inc.). It allows an external examiner to judge retinal image quality at each step of the refraction process—from the fogging technique [1,2] to the cylindrical component estimation and the final adjustment of the sphere. Fig 1 shows the software’s graphical user interface (GUI).

In order to computer-generate retinal images, a reduced schematic eye [12] having a focal length of 22.4 mm and a refractive index of 1.33 is assumed as starting point. Each patient’s specific aberrometry data can be then added to this schematic eye model.

The software includes a virtual phoropter to simulate the adding of spherical and cylindrical lenses in front of the eye and the possibility to flip and rotate a Jackson’s Cross Cylinder lens.

The retinal image is then calculated by combining the patient’s aberrometry data, the lenses added with the phoropter and the JCC lens with the schematic eye model.

To compute the retinal image, the point-spread function (PSF) is first calculated as the intensity of the complex pupil function P(x, y)’s Fourier transform (ℱ):

$$PSF\left(x,y\right)={\left|F(P\left(x,y\right)\right|}^{2}={\left|F\left(p\left(x,y\right)e^{-i\frac{2\pi }{\lambda }W_{T}\left(x,y\right)}\right)\right|}^{2}$$ (1)

Where (x,y) are transverse coordinates in the eye’s exit pupil plane, λ is the wavelength (587 nm) and W_T_(x,y) is the total phase:

$$W_{T}\left(x,y\right)=(W_{eye}\left(x,y\right)+W_{lens}\left(x,y\right)+\left[W_{JCC}\left(x,y\right)\;*\;S\left(x,y\right)\right])$$ (2)

Where W_eye_(x,y) is the phase for the reduced schematic eye together with the patient’s aberrometry data, W_lens_ (x,y) represents the phase of the lens that we place in the virtual phoropter and W_JCC_ (x,y) represents the JCC test’s cross-cylinders. Before calculating phase functions W_lens_ (x,y) and W_JCC_ (x,y), a conversion of the lenses power—from the virtual phoropter’s plane to the exit pupil plane [1]—was carried out so as to compute all phase functions in the same plane. S(x,y) is a Heaviside step function whose value is zero when the JCC test is deactivated and one when the JCC test is activated.

**Fig 2:**
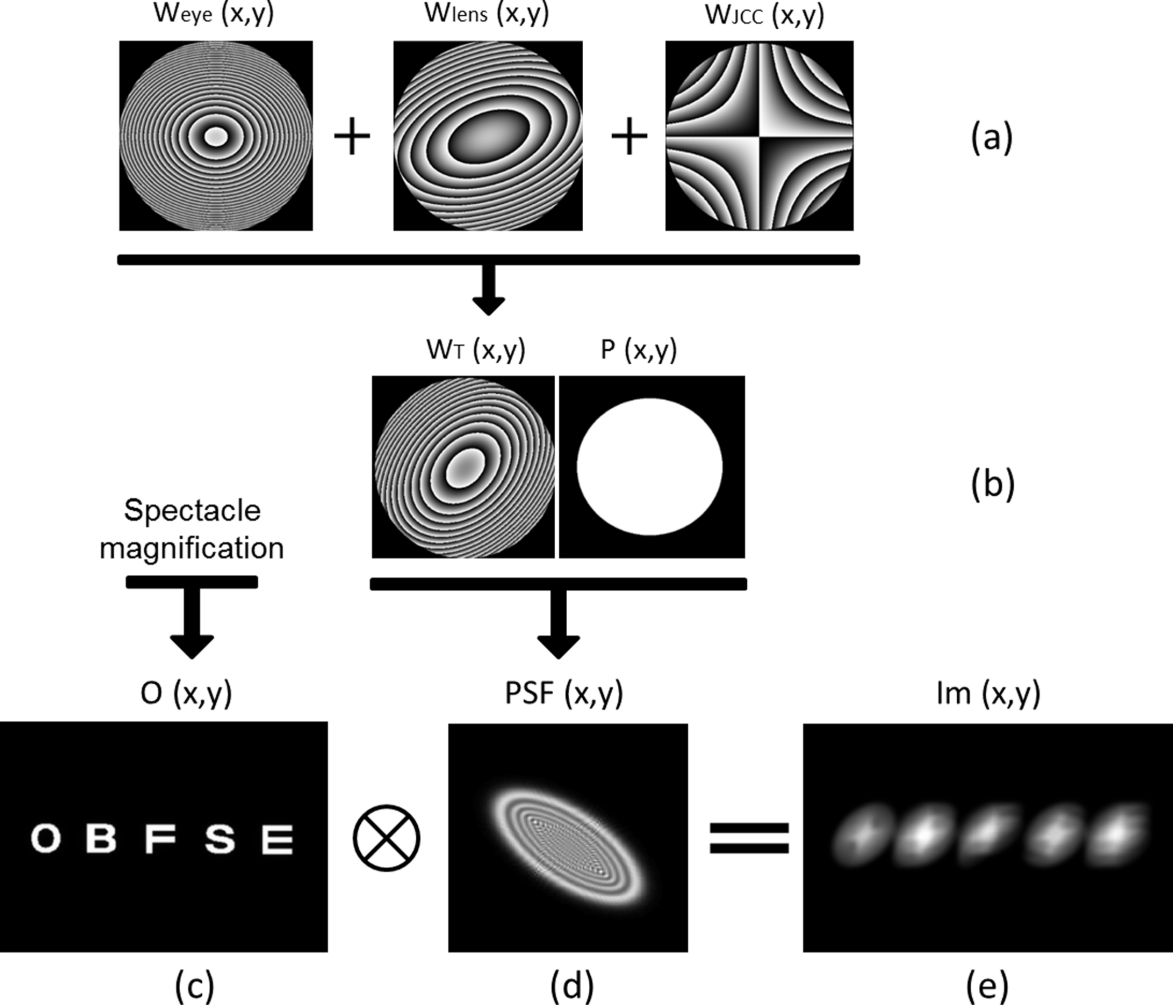
Full process for retinal image computation. Scheme of the procedure followed to generate the retinal image of a line of letters (0.20 logMAR). (a) Phase functions corresponding to the artificial eye (W_eye_), the phoropter’s lens (W_lens_) and Jackson’s Cross Cylinder test lens (W_JCC_) (b) Sum of the three phase functions (W_T_ (x,y)) and pupil function (P(x,y)), (c) Paraxial image affected by spectacle magnification, (d) PSF obtained in step (b) and (e) Retinal image, resulting from the convolution of the paraxial image and the PSF. Cross-cylinder power is ±0.50 D. Pupil radius is assumed to be 3 mm.

Secondly, the PSF and the paraxial image of a given optotype O(x,y) are convoluted to obtain the retinal image (Im (x, y)). Fig 2 illustrates the full process of retinal image computation.

The anamorphic distortion is taken into account in the paraxial image before proceeding with the convolution step so as to ensure realistic conditions. This distortion effect is due to spectacle magnification (SM) and refers to the change in an object’s apparent size when a lens is placed in front of the eye [13]. There are studies in the literature regarding this effect [14] but it has never been included nor mentioned in the context of simulation of refraction methods.

In our software we assume that the lenses we add by means of the virtual phoropter are thin lenses, which implies that SM can be computed using the following expression:

$$SM=1/\left(1-d_{v}F\right)$$ (3)

where d_v_ is the distance from the lens to the entrance pupil and F is the lens power in dioptres. In the case of sphero-cylindrical lenses SM has to be calculated for both main meridians.

This method allow us to compute the retinal image of a range of optotypes (line of letters, single letter, Snellen chart, astigmatic dial, etc.) having different sizes.

The software also offers the possibility to change pupil size (entrance pupil). The program recalculates the Zernike coefficients for the eye aberrometry data for the new pupil radius and yields a new retinal image. Moreover, the software allows the user to select or modify each individual Zernike aberration coefficient, which is especially useful to assess the influence of a specific aberration upon retinal image quality.

Besides the phoropter and Jackson’s Cross Cylinder test, the software offers the possibility to include the contribution (i.e., the phase) corresponding to any other visual compensation; for example, a multifocal contact lens. Besides providing the retinal image, the software also computes various related visual metrics [8,9] (among them, Visual Strehl’s ratio) so as to have an objective criterion to evaluate retinal images.

The next section shows several application examples of this simulation software.

## Results and Discussion

### Clock dial test to determine the presence of astigmatism

The clock dial test is now a routine activity that helps the eye care professional to estimate the astigmatism’s magnitude and orientation. The test consists of a circular chart (angular size of 250 arc minutes) with radii drawn at 30° intervals. The patient has to identify first the sharpest line and then the optometrist has to add cylindrical lenses of varying power oriented at a specific angle until all the radii appear equally clear.

**Fig 3:**
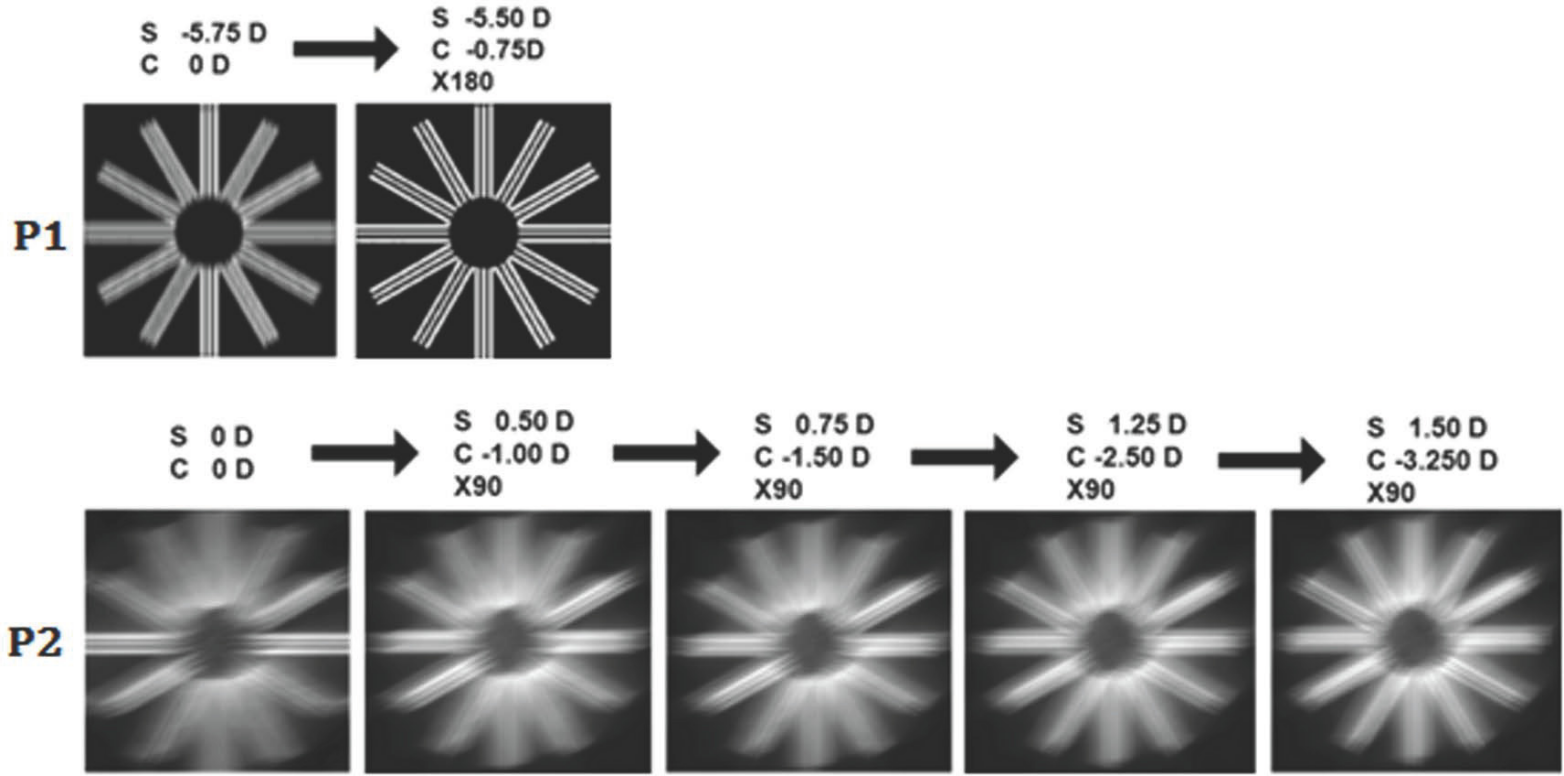
Simulated retinal images for a clock dial test. Retinal images of a clock dial test computed for two subjects (P1 and P2) having very different aberration patterns. The images on the left (i.e., the first one of each row) correspond to the simulated retinal image when only spherical compensation has been virtually added. The remaining images correspond to the addition of the amount of cylinder power shown on top of each image, until equality was achieved. Pupil radius was 2 mm in all cases.

This clock dial test is generally performed at the beginning of the subjective-refraction process for far vision, once the optometrist has added enough spherical compensation to reach 0.50 logMAR. Fig 3 shows test’s simulated results for two different subjects: subject P1 (top), who has only low-order aberrations (LOA) and subject P2 (bottom), who has irregular astigmatism and an RMS_HOA_ = 1.44 μm (S1 Table). Pupil radius was 2 mm for both subjects.

For this example, each simulated image was computed based on a total phase W_T_(x,y) given by Eq 2, where W_eye_ (x,y) represents either subject P1’s or P2’s aberration pattern and W_lens_ (x,y) corresponds to different sphero-cylindrical lenses (S,C Xα), as detailed on top of each image.

For instance, if we examine P1’s simulation in Fig 3 (top), we can see that with no astigmatism compensation (first image) there is a clear candidate for the sharpest line (i.e., the vertical line), and equality can be then clearly reached by adding a cylinder lens of -0.75 D @180°. Contrariwise, for P2, even though it is possible to identify the sharpest line (i.e., the horizontal line), it would not be easy for the subject to know which cylindrical lens power is best to reach sharpness equality. Astigmatic dials used in subjective refraction process frequently lead to confusing answers by the patients. In this context, simulation software is a useful method to gain a deeper insight into the difficulties that they encounter.

### Jackson’s Cross-Cylinder (JCC) test

In the context of step-wise subjective refraction, Jackson’s Cross-Cylinder (JCC) test is the main technique that is used to measure the power and axis of the cylindrical correction.

**Fig 4:**
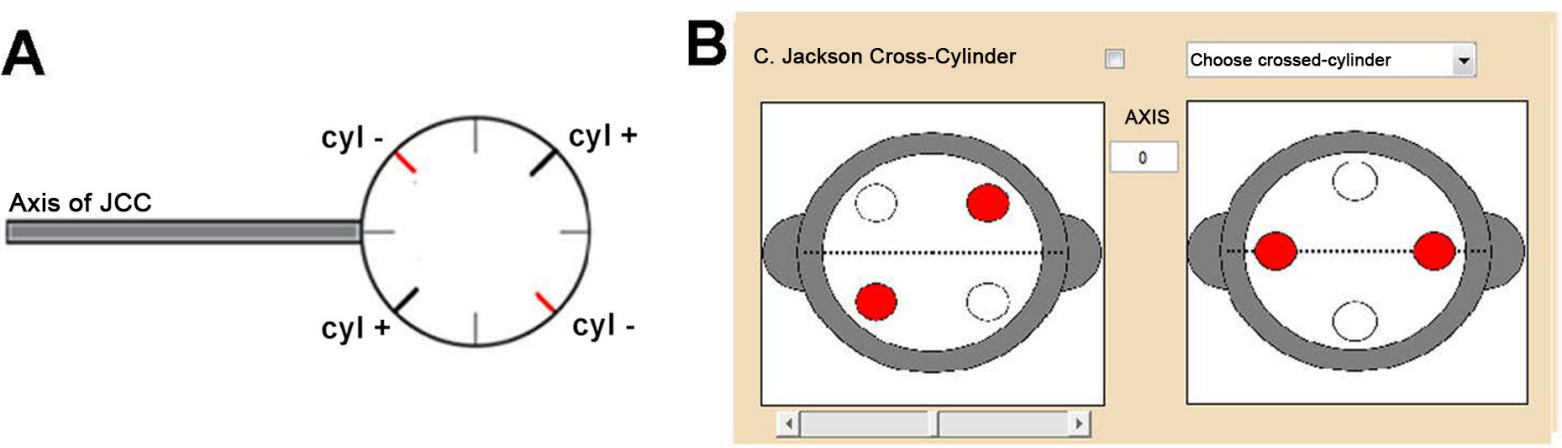
Jackson’s Cross Cylinder. (A) A typical Jackson’s Cross Cylinder. Red lines indicate negative (minus) cylinder and black lines indicate positive (plus) cylinder. The handle points out the cross cylinder’s axis. (B) Section of the simulation software interface corresponding to Jackson’s Cross-Cylinder configuration. The pictures are used to determine the cylindrical correction axis (right) and power (left).

In 1887, Edward Jackson [15] described the use of a fixed-power Stokes lens to determine the cylinder power that was needed to correct astigmatism. Then, in 1970, he indicated that this lens could also be used to obtain the correcting cylinder axis [16]. As shown in Fig 4A, the cross cylinder is a combination of two cylinders of equal power but opposite signs whose axes are perpendicular to each other.

In their standard practise the eye-care professional flips the lens in front the patient’s visual axis. Firstly, in order to determine the orientation of the astigmatic compensation, the cross cylinder axes must be placed parallel to the axis of the compensation we are going to introduce. The cross cylinder flips around its axis and the patient compares the two blurred images and chooses the best one. The cross cylinder is then rotated until both images are as similar as possible. Next, in order to determine the power of the astigmatic compensation, the cross cylinder axes must be at 45° with respect to the compensation axis previously obtained. Again, the cross cylinder flips and the patient has to compare the two blurred images and choose the best one. The cylindrical compensation’s power is gradually increased until image equality is attained. In both cases, the end-point of the procedure has been reached and the astigmatism’s axis and power has been identified when similar sharpness (or blurring) is achieved.

**Fig 5:**
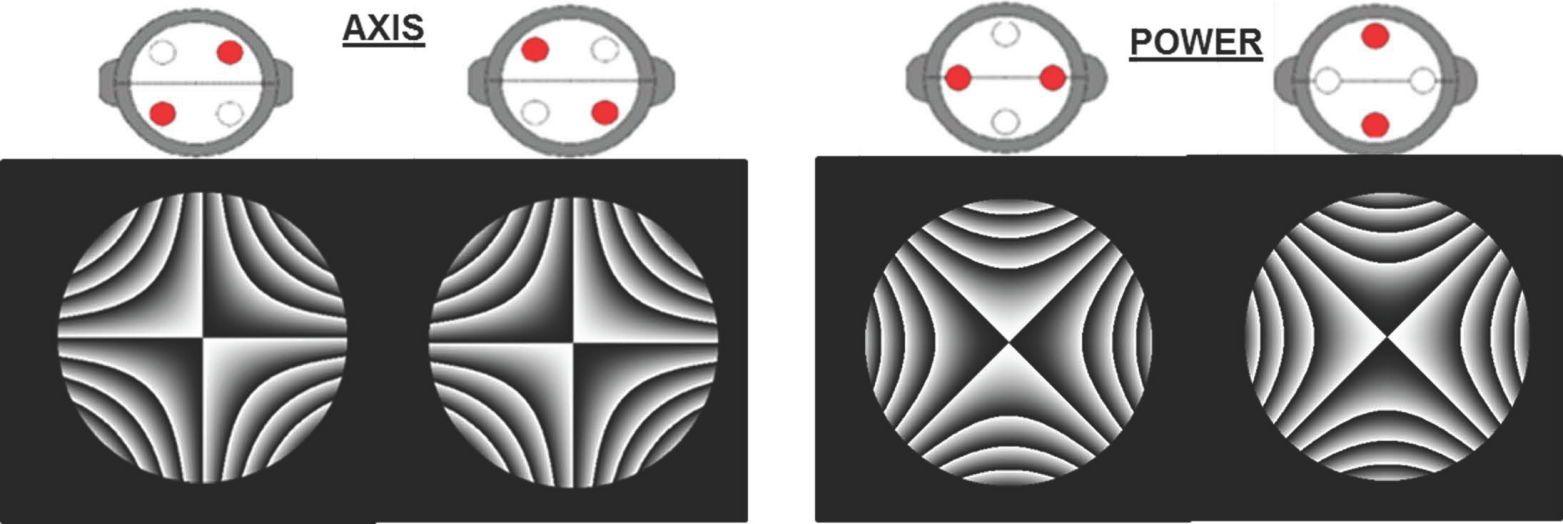
Interferometric images of Jackson’s Cross Cylinders. Interferometric images shown in the simulation software representing the phase function for each position of the Jackson’s Cross-Cylinder (JCC). The image on the left is used to determine the cylindrical correction’s axis and the image on the right is used to determine the cylindrical power. Cross cylinder power is ±0.50 D. Pupil radius was 3 mm.

The software interface shows two pictures of the cross cylinder (Fig 4B); each one of them is related to one of the procedures described above: determination of the astigmatic correction axis (left) and power (right). The program virtually flips the cross cylinder when the appropriate picture is clicked. Interferometric images representing the phase function corresponding to each JCC position are shown in Fig 5. The simulation software interface allows the user to decide whether or not they want to use the JCC test, as well as to choose the specific power of the cross cylinder lens.

When we begin the JCC test, the circle of least confusion (CLC)—i.e. the dioptric midpoint between the anterior and posterior focal lines—must be positioned on the retina of the subject [17]. This is an important requirement and, in order to fulfil it, throughout the test a +0.25 D sphere is added for each -0.50 D of cylinder power addition.

**Fig 6:**
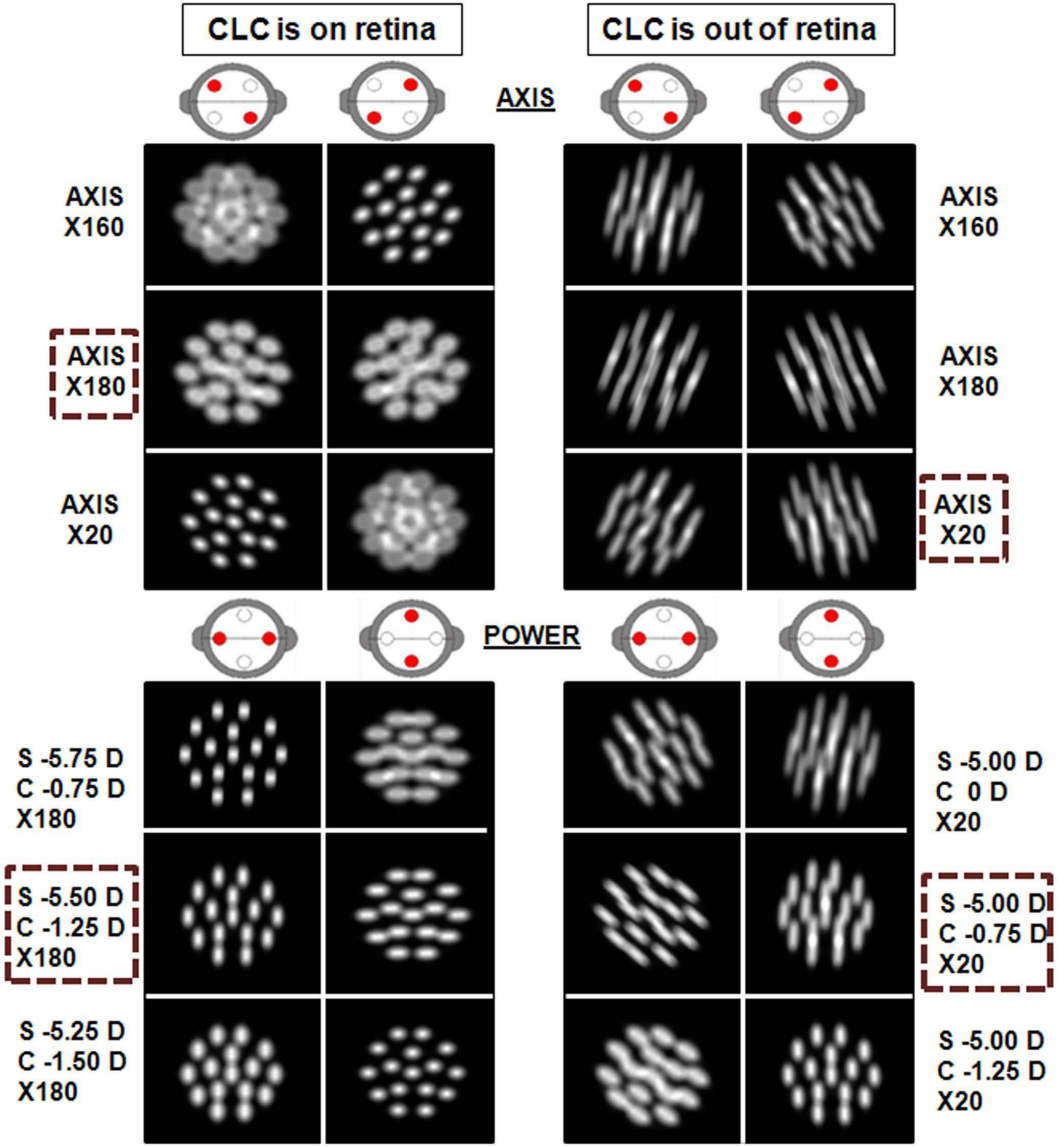
Simulated retinal images during JCC with a dot pattern. Retinal images with a dot pattern as chart. On the left side, JCC test is performed while maintaining the CLC on the retina. On the right side, JCC test when CLC is placed elsewhere. The selected sphero-cylinder, once the compensation axis and cylindrical power had been determined, is indicated with a dashed line. Cross cylinder power is ±0.50 D. Pupil radius is 2 mm.

Our simulation software allows us to demonstrate what happens when the CLC is not placed on the retina throughout the test. For the subject P1 (S1 Table), Fig 6 shows two different procedures: JCC test while maintaining the CLC on the retina (left column) and JCC test when the CLC is elsewhere (right column). In both cases a cross-cylinder with ±0.50D power and a dot pattern (angular size of 50 arc minutes) was used.

Fig 6 shows that equality can be easily identified when the CLC was on the retina, but when the CLC fails to fall on the retina the comparison becomes more difficult for the patient; i.e., any one of the three options showed in Fig 6 could have been an acceptable candidate when determining the compensation axis, which can lead to different final sphero-cylinder compensation.

**Fig 7:**
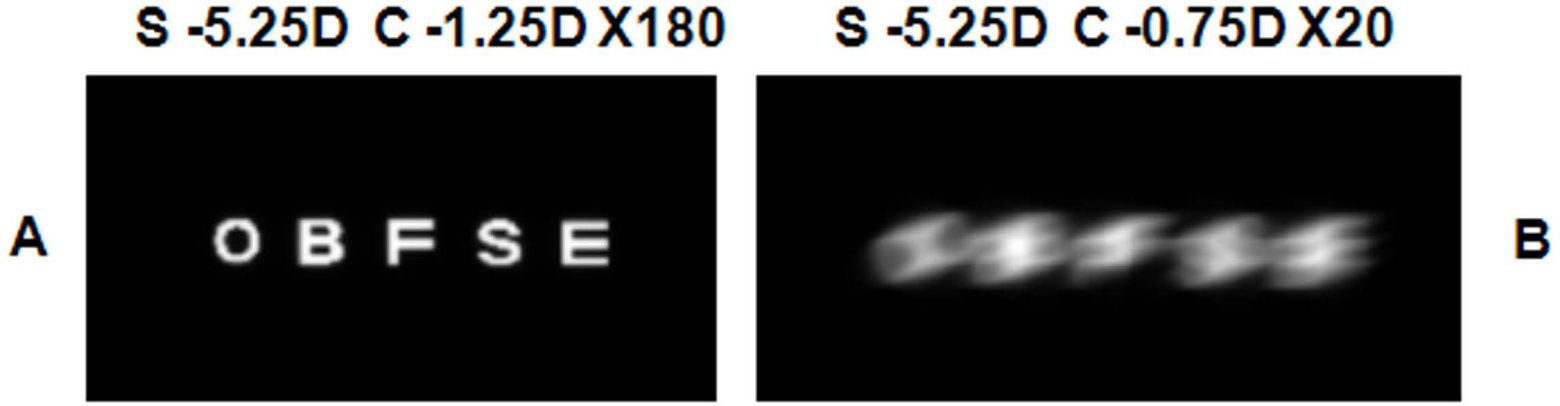
Simulated retinal images with the best compensation yielded by a JCC test where a dot pattern was used as chart. Retinal images of a line of letters (0 logMAR) with the best sphero-cylindrical refraction that the JCC test yielded in two different scenarios: the CLC was maintained on the retina (A) and the CLC position was not closely controlled (B). Pupil radius was 2 mm.

To further illustrate this fact, Fig 7 shows the simulated retinal images (0 logMAR) for the best sphero-cylindrical compensation yielded by the JCC test, for both CLC positions described above after adjusting the spherical power. As could be expected, the resulting visual quality is significantly better in the scenario where the CLC was maintained on the retina throughout the JCC test.

Apart from the CLC position, the chart used during the test could influence the resulting sphero-cylindrical compensation. For this reason, some authors suggest that a chart comprising a dot pattern is preferable [17].

**Fig 8:**
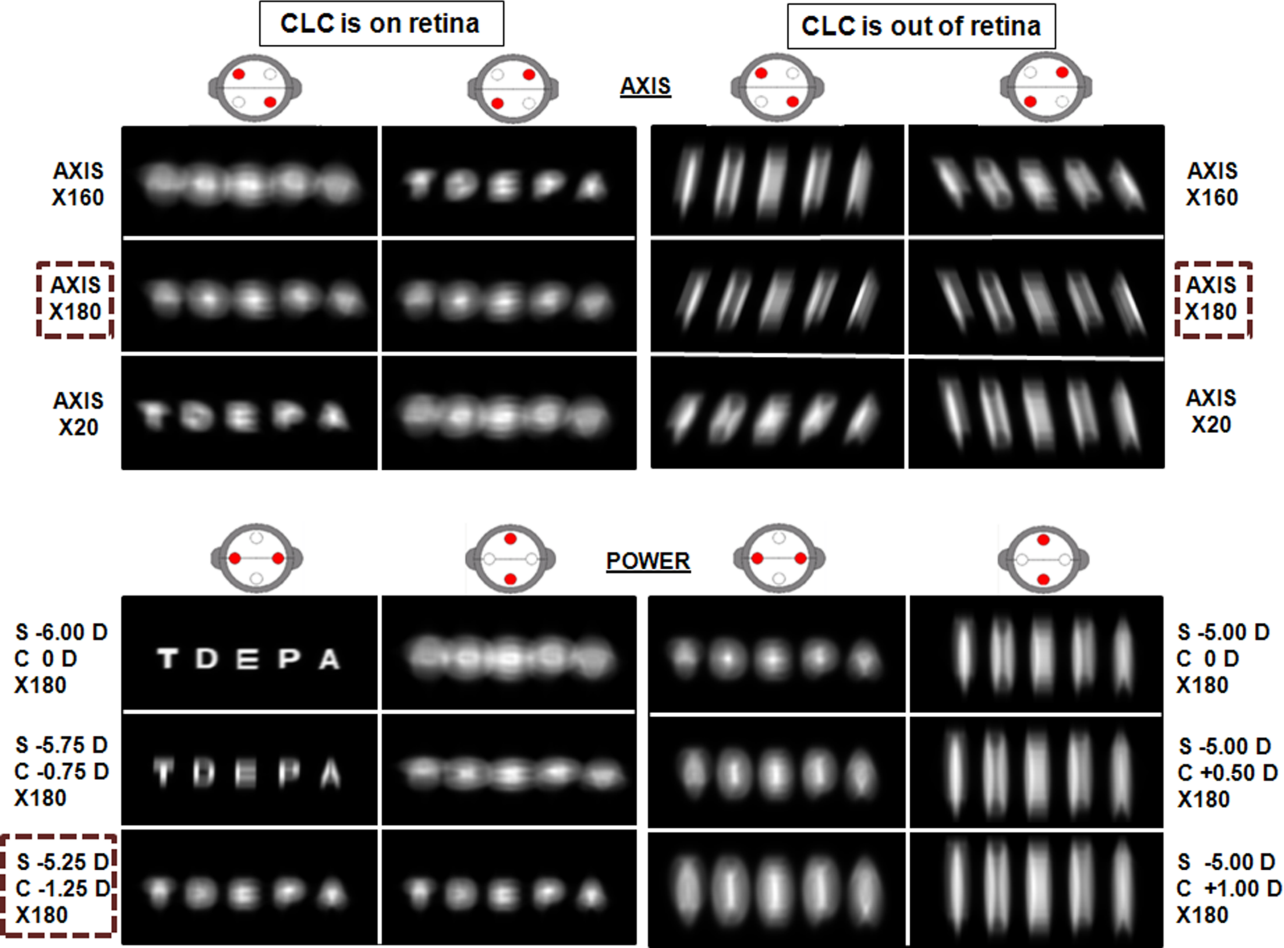
Simulated retinal images during JCC test with a line of letters. The selected sphero-cylinder, once the compensation axis and cylindrical power had been determined, is indicated with a dashed line. Cross cylinder power is ±0.50 D. Pupil radius was 2 mm. Visual acuity for the line of letters corresponds with 0.20 logMAR.

In this context, Fig 8 shows the simulated retinal images using a line of letters (0.20 logMAR) as chart in the same patient and scenarios as described in the context of Fig 6: JCC test with the CLC being maintained on the retina (left column) and a scenario where the CLC is placed elsewhere (right column)

As can be inferred from Fig 8, again when the CLC lies on the retina it is easy to identify for which particular pair both images were equally blurred. However, when the CLC is out of the retina it becomes impossible to reach a situation in which both images were equally blurred, so determining the optimum cylinder power becomes very difficult, and the final result depends clearly on the person who judges the images.

**Fig 9:**
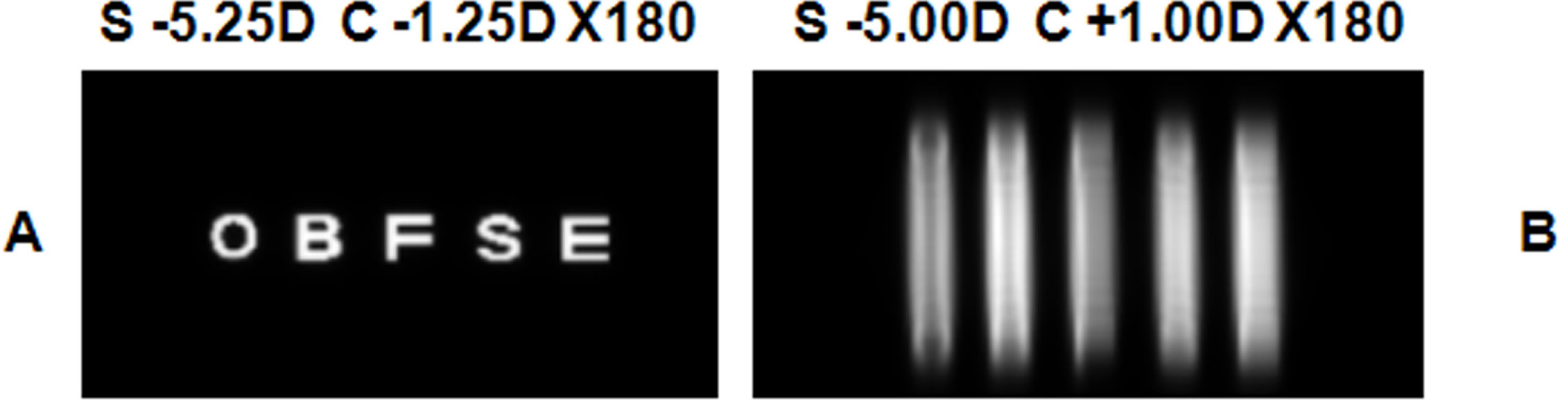
Simulated retinal images with the best compensation yielded by a JCC test where a line of letters was used as chart. Retinal images of a line of letters (0 logMAR) with the best sphero-cylindrical refraction that the JCC test yielded in two different scenarios: the CLC was maintained on the retina (A) and the CLC position was not closely controlled (B). Pupil radius was 2 mm.

Fig 9A shows the simulated retinal images for a line of letters (0 logMAR) for the best sphero-cylindrical compensation yielded when the CCJ test is performed with a line of letters maintaining CLC on the retina. As it can be seen in Fig 8, there is not any sphero-cylindrical compensation that provides two images equally blurred in the case of placing CLC out of the retina, so we have considered one of the sphero-cylindrical compensations obtained with the CCJ test performed with a line of letters to simulate the retinal image in Fig 9B. Visual quality is better when the CLC is on the retina throughout the CCJ test, as happened when the CCJ test was performed with a dot pattern (Fig 7). Besides, if we compare images in Fig 7B and Fig 9B, we found that the image obtained with the CLC out of the retina is worse when the CCJ test is performed with a line of letters.

Previous empirical work by Sims [4] shows that equal blur cannot always be achieved and that there are many factors that could affect the final decision. The results yielded by our virtual refraction software confirm the influence of JCC test’s CLC position on the final compensation. Besides, the software helps us to highlight the influence of the specific pattern chosen as chart when the CLC is not placed exactly on the retina.

Nevertheless it also is important to highlight that this result is only applicable to patients having negligible high-order aberration, such as P1. A real eye, however, is not a perfect system and high-order aberrations result in an asymmetric Sturm interval, which increases the difficulty of placing the CLC exactly on the retina.

As far as we know, it is the first time that retinal images produced during a cross-cylinder test are shown, allowing the external examiner to get an idea of the images seen by their patients.

### Performance analysis of multifocal contact lens designs

As mentioned before, simulation software offers the possibility to introduce a phase plate together with the patient’s aberrometry data and phoropter lenses, allowing us to test new designs for vision correction; for instance, multifocal contact lenses.

We analyse two concentric-ring multifocal contact lens designs in which each lens’ annular regions are designed for either distance, intermediate or near correction. Each contact lens’ phase profile is fed into the simulation software.

**Fig 10:**
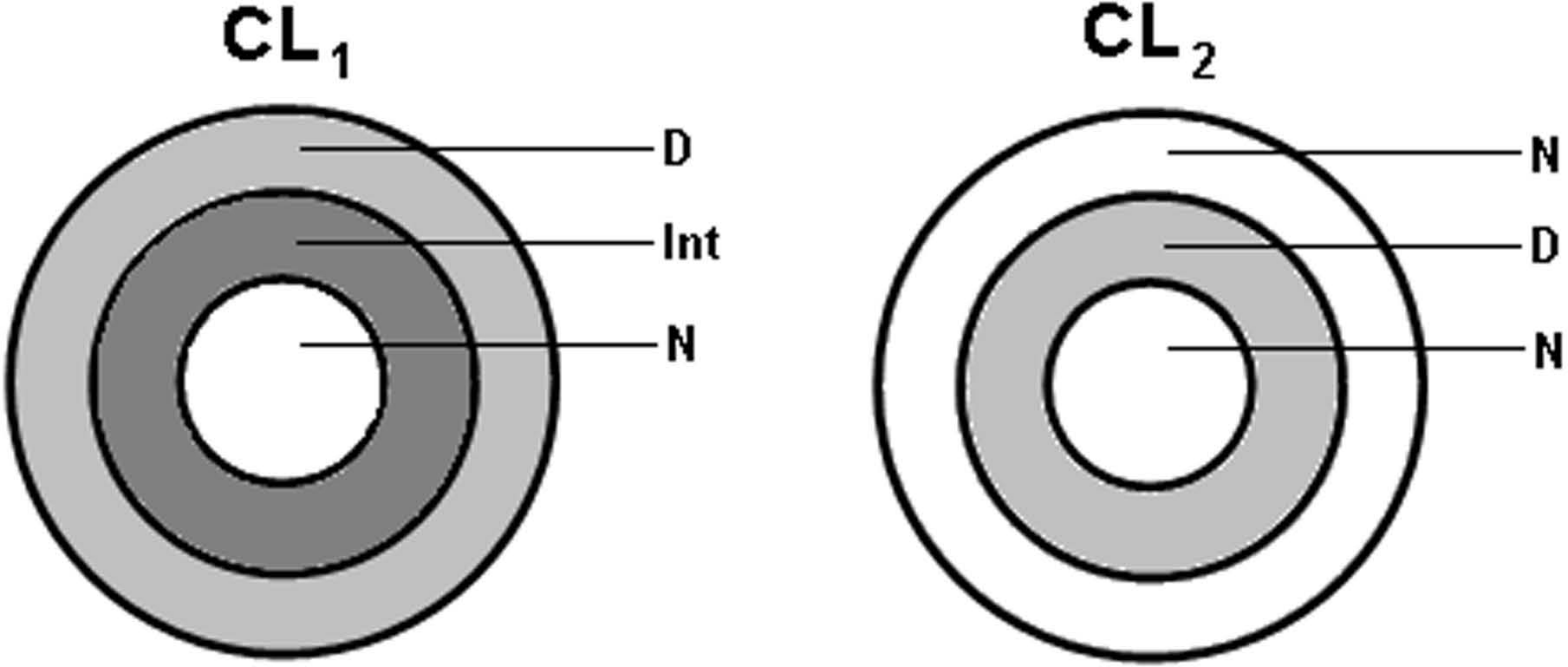
Multifocal concentric-ring contact lens designs. CL_1_ is a centre-near (N) bifocal design contact lens with a distance zone (D) and intermediate-vision power (Int). CL_2_ is a centre-near bifocal design contact lens with no intermediate-vision zone.

Fig 10 shows both designs’ (CL_1_ and CL_2_) main features. CL_1_ is a centre-near bifocal design contact lens with three rings: an external distance-vision (D) zone and a central near-vision (N) zone separated by a transition (Int) zone having +1.00 D for intermediate vision. CL_2_ is a centre-near bifocal design contact lens with three rings alternating distance (D) and near (N) prescription. Each contact lens design has +2.00 D of add power and has been evaluated for an emmetropic and aberration-free eye. We assume that the contact lens covers the whole pupil area.

**Fig 11:**
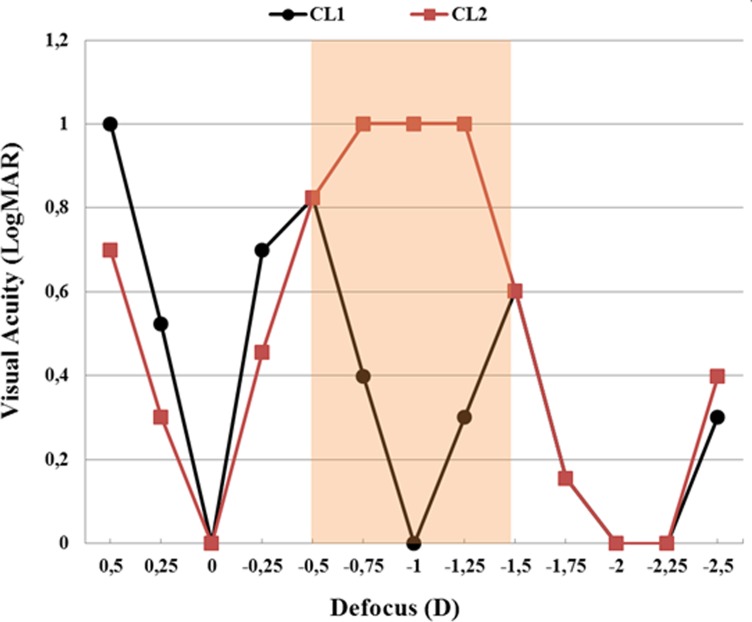
Defocus curves. Defocus curves for CL_1_ (black dots) and CL_2_ (red squares) multifocal contact lenses. The shaded area corresponds to the intermediate-vision zone. Pupil radius was 3 mm.

Fig 11 shows the defocus curves yielded by the simulation software for each contact lens design (CL_1_ and CL_2_), where visual acuity (in logMAR) is computed as a function of the defocus induced by means of virtual spherical lenses. As could be expected, CL_2_ does not provide good VA for intermediate vision (shaded zone), where VA goes up to 1 logMar (i.e., it falls down to 0.1 in a decimal scale).

**Fig 12:**
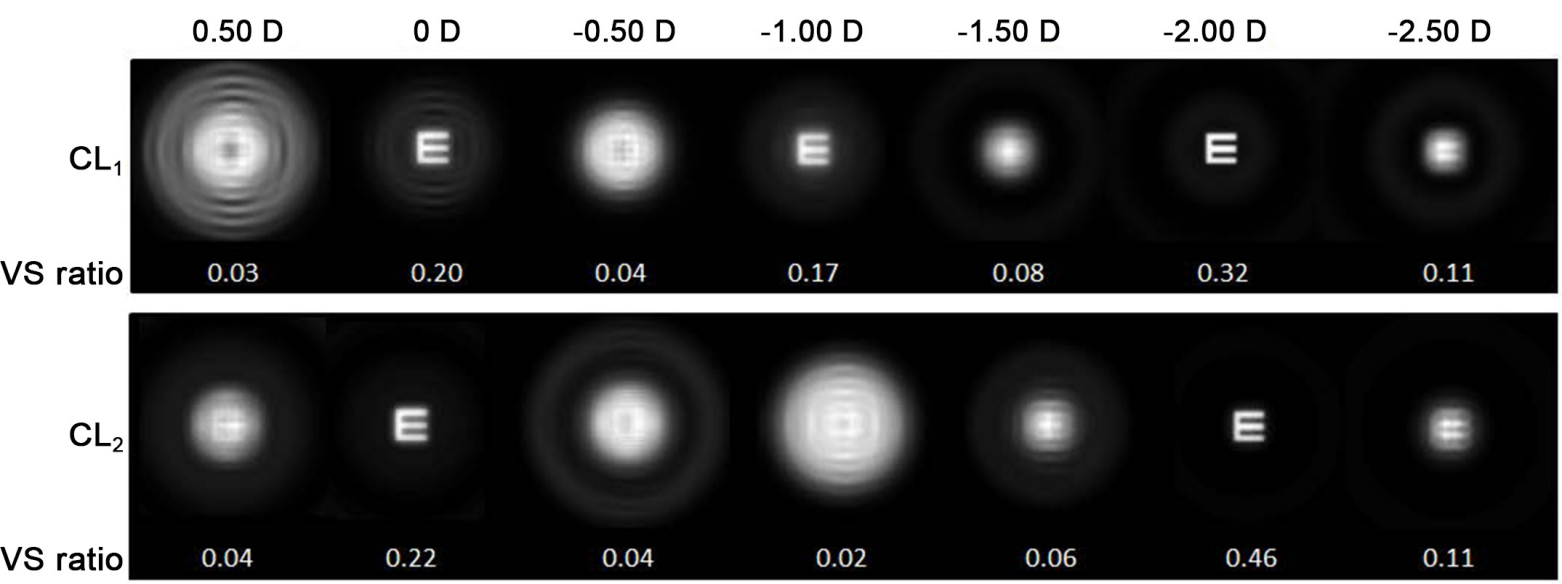
Through-focus simulated retinal images yielded by contact lens designs CL_1_ and CL_2_. Retinal images obtained with CL1 and CL2, corresponding to a Snellen’s E with 0 logMAR located at different distances from the eye, going from more distant objects (left) to closer ones (right). The amount of defocus (in diopters) is indicated at the top of the figure. VS ratio is shown under each image. Pupil radius was 3 mm.

To illustrate the range of sharp vision that each design provides, we computed (see Fig 12) the set of retinal images corresponding to the defocus curves above, for a 0 logMAR Snellen’s E. To be able to objectively assess these designs’ performance we resorted to an image metric which correlates well with the quality of the perceived images; i.e., the Visual Strehl (VS) ratio [18], whose value for each retinal image is also shown in Fig 12.

Regarding VS ratio, the values obtained with both designs are relatively high for far and near distances, but it is worth pointing out that CL_1_ provides better visual quality and higher VS for intermediate vision.

Generally speaking, the JCC technique is not to be recommended when assessing multifocal contact or intraocular lenses, due to their particular optical characteristics. The limitation of conventional refractive procedures in eyes with multifocal lenses has been studied elsewhere [19,20], but retinal image quality during those processes has never been analysed. For this reason, we want to show for the first time what happens when a JCC test is performed on a multifocal contact lens wearer.

**Fig 13:**
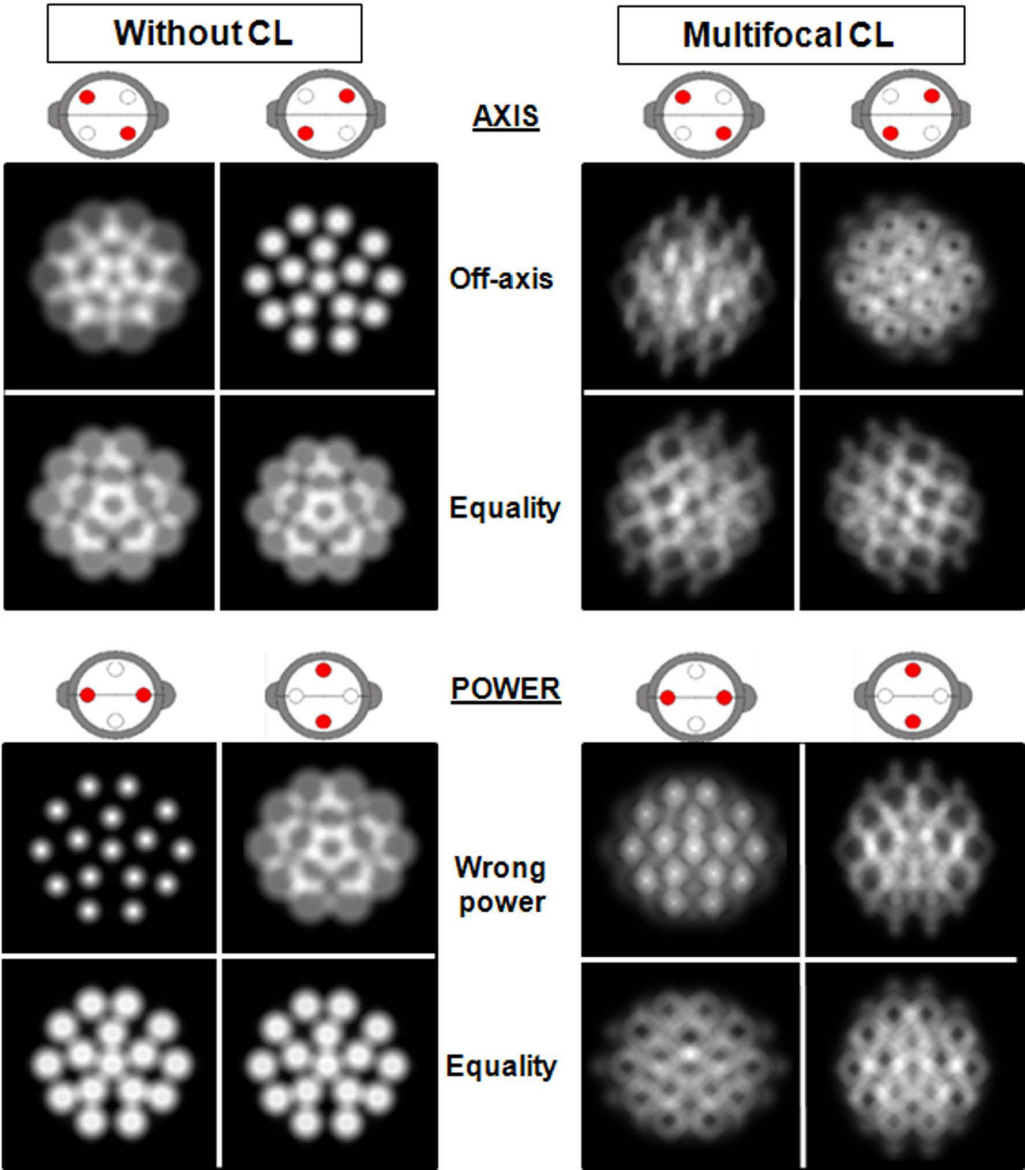
Simulated retinal images during a Jackson’s Cross Cylinder test with and without a multifocal contact lens. Simulated retinal images produced during a JCC test for a subject wearing a multifocal contact lens (CL) (right column) or without CL (left column). Cross cylinder power was ±0.50 D and pupil radius was 3 mm.

In Fig 13, we simulated the retinal images of a dot pattern that would be produced during a JCC test for a subject having no high-order aberrations and -0.75 D of residual astigmatism under two scenarios: wearing no contact lens and with CL1’s multifocal contact lens.

Halo or blurring effects associated with the multifocal ring lenses complicate the election between pairs of images. Both image pairs are too blurred and equality is hard to be detected. Contrariwise, when the same subject is wearing no contact lens—similarly to what we saw earlier—there is no doubt about for which image pair the two images are most similar.

## Conclusions

A novel simulation software of subjective refraction based on the computation of retinal images is presented. As we have demonstrated in the present paper for the specific case of the JCC test, this software can help to gain a deeper insight and to improve different refraction techniques. In particular, it has shown the importance of maintaining the circle of least confusion on the retina during the whole duration of the JCC test. As far as we know, this is the first work where, in the context of a JCC test, retinal images for different charts have been shown. The observation of these images has pointed out the importance of using a dot pattern—instead of a line of letters—in a JCC test.

This software has also been used to compare the simulated image quality for two different multifocal contact lens designs and, furthermore, it has allowed us to show what happens when the JCC test is used to assess a multifocal-contact-lens wearer.

Nevertheless, in its current form, this software also has some limitations that cannot be ignored. First, the software does not take into account chromatic aberration, pupil dynamics or eye accommodation, and nor does it consider the sampling of the cone mosaic or neural processing [21,22,23]. These improvements will be addressed in future research studies in order to increase the quality of this simulation software.

## Supporting Information

S1 Table Zernike coefficients in the OSA standard ordering from subjects P1 and P2. (XLSX)
